# The Effects of an Educational Intervention About Front-of-Package Labeling on Food and Beverage Selection Among Children and Their Caregivers: Protocol for a Randomized Controlled Trial

**DOI:** 10.2196/54783

**Published:** 2024-04-01

**Authors:** Diana Avila-Montiel, Jenny Vilchis-Gil, América Liliana Miranda-Lora, Lubia Velázquez-López, Miguel Klünder-Klünder

**Affiliations:** 1 Epidemiological Research Unit in Endocrinology and Nutrition Hospital Infantil de México Federico Gómez CDMX Mexico; 2 Clinical Epidemiology Research Unit Dr. Carlos Mac Gregor Sánchez Navarro Mexican Social Security Institute CDMX Mexico

**Keywords:** e-Health nutrition education, ultraprocessed foods, malnutrition, children, Mexico, intervention, obesity, food, food selection, labeling, package labeling

## Abstract

**Background:**

Overweight and obesity pose a global public health challenge and have a multifactorial origin. One of these factors includes obesogenic environments, which promote ultraprocessed foods characterized by being high in calories, saturated fats, added sugars, and sodium. In Mexico, it has been estimated that 30% of the total energy consumed comes from processed foods. The Modification to the Official Mexican Standards introduces nutritional information through black octagonal seals that alert consumers about products with excessive amounts of some components for a better food selection in the population. However, the effects of warning labels on processed food selection and purchases among children remain unknown.

**Objective:**

We aimed to evaluate the impact of a digital educational intervention focusing on front-of-package warning labels on the food selection and purchasing behavior of elementary schoolchildren and their caregivers.

**Methods:**

Children from 4 elementary schools in Mexico City, 2 public and 2 private schools, will participate in a randomized controlled trial. The schools will be chosen by simple random sampling. Schools will be randomized into 2 groups: intervention and control. In the control group, the dyads (caregiver-schoolchildren) will receive general nutritional education, and in the intervention group, they will receive guidance on reading labels and raising awareness about the impact of consuming ultraprocessed products on health. The educational intervention will be conducted via a website. Baseline measurements will be taken for both groups at 3 and 6 months. All participants will have access to an online store through the website, allowing them to engage in exercises for selecting and purchasing food and beverages. In addition, other measures will include a brief 5-question exam to evaluate theoretical understanding, a 24-hour reminder, a survey on food habits and consumption, application of a food preference scale, anthropometric measurements, and recording of school lunch choices.

**Results:**

Registration and funding were authorized in 2022, and we will begin data collection in September 2024. Recruitment has not yet taken place, but the status of data analysis and expected results will be published in April 2025.

**Conclusions:**

The study is expected to contribute to evaluating whether reinforcing front-of-package warning labels with education enhances its effects and makes them more sustainable. Conducting this study will allow us to propose whether or not it is necessary to develop new intervention strategies related to front-of-package labeling for a better understanding of the population, improved food choices, and better health outcomes.

**Trial Registration:**

ClinicalTrials.gov NCT06102473; https://clinicaltrials.gov/study/NCT06102473

**International Registered Report Identifier (IRRID):**

PRR1-10.2196/54783

## Introduction

Overweight and obesity pose a global public health challenge. In Mexico, data from the National Health and Nutrition Surveys from 2012, 2018, and 2020 show that the combined prevalence of overweight and obesity in children between the ages of 5 and 10 years has remained high (33.2%, 35.6%, and 38.2%, respectively) [1].

Obesity has a multifactorial etiology, involving genetic, environmental, and behavioral aspects. Obesogenic environments promote ultraprocessed foods, which are high in calories, saturated fats, added sugars, and sodium [2,3].

Therefore, public policies aimed at reducing the incidence and prevalence of childhood obesity must consider actions that modify the environment to improve nutrition. Labeling of ultraprocessed products has been a common strategy that governments worldwide use to improve people’s diets [4].

In Mexico, it has been estimated that more than 30% of the total energy consumed comes from ultraprocessed foods, favoring the prevalence of overweight and obesity. The daily dietary guidelines by the Ministry of Economy and the Ministry of Health in Mexico were an effort for people to improve their food selection and purchasing; however, they did not show good results due to the complexity of interpreting them [5].

The Modification to the Official Mexican Standard (NOM-051-SCFI/SSA1-2010 [NOM 051]) was approved in 2020. This proposed a front labeling system for foods and beverages that consisted of nutritional information through black octagonal seals, with legends like “excess calories,” “excess sodium,” “excess saturated fat,” “excess trans fat,” “excess sugars,” and other explanations, such as “contains caffeine, avoid for children” and “contains sweeteners, not recommended for children,” to provide information to the consumers to help them make food selections [5,6].

Ultraprocessed products targeted at children are offered in all supermarkets and represent an important commercial income for the food industry [7,8]. At home, parents are primarily responsible for purchasing food and have a significant influence on their children’s eating behaviors [7-10].

On the other hand, children also influence parents’ purchasing decisions, especially when they want to choose unhealthy foods, which is why the influence of front labeling must be studied from both perspectives (ie, children’s and parents’) [11]. To reduce the purchase and consumption of ultraprocessed foods, various marketing strategies have been implemented, especially for products aimed at school children; however, the results have been varied and limited [12,13].

It is known that food packaging has an impact on how children perceive these products. For instance, the use of drawings and bright colors can make them associate the product with something suitable to eat, which is why changes have been made to packaging designs to discourage consumption, mainly by removing cartoon slogans or legends that promote their consumption [14].

Some types of front-of-package labeling guide the daily intake by indicating the proportion (%) of nutrients found in a serving according to the recommended daily intake for adults. This includes the traffic light system, which uses colors (green, yellow, and red) to indicate the amount of nutrients in a product, as well as warning labels, which are black octagonal symbols, which indicate the excess of nutrients, energy, and substances present in the product and are intended to discourage purchase and consumption [15].

Having identified the relationship between packaging and food consumption, it is expected that front-of-package nutritional labels will reduce the consumption of unhealthy foods [16].

Furthermore, it has been documented that another effective strategy to improve food selection is to enhance nutritional education to develop skills to distinguish healthy products from those that are not considered healthy [17].

Mobile nutritional interventions have been observed to reach a broader range of social and demographic groups and can facilitate the selection of healthy foods at the point of purchase, optimizing decision-making times [18].

In Mexico, it is estimated that 78.6% of the Mexican population aged 6 years and older has access to the internet, with 97% connecting through mobile devices. This is why various health institutions have implemented web portals, social networks, and the use of cell phone messages, telephone, and mobile apps for managing, preventing, and treating health-related activities [19-21].

The objective of this clinical trial is to evaluate the impact of a digital educational intervention focusing on front-of-package warning labels on the food selection and purchasing behavior of elementary school children and their caregivers [22].

## Methods

### Recruitment and Study Design

Third-, fourth-, and fifth-grade elementary school students and their caregivers from 4 primary schools—2 public and 2 private schools—in Mexico City will participate in the study. The schools will be chosen by simple random sampling. Schools will be randomized into 2 groups: intervention and control.

After approval from the directors of the primary schools, meetings will be held with parents to invite them to participate; they will be explained the intervention’s objectives, activities, and duration. Parents and schoolchildren will be invited to sign a consent and informed assent, respectively, clarifying that their participation is voluntary, and they may choose to discontinue at any time without affecting their activities at school.

The main question this study aims to answer is the following: What is the effect of a digital educational intervention about front-of-package warning labels on food selection among children attending primary schools in Mexico City, compared to a control group?

Schools will be randomized into 2 groups. In the control group, the dyads (caregiver-schoolchildren), will receive general nutritional education, and in the intervention group, they will also receive guidance on reading labels, which raises awareness about the impact of consuming processed or ultraprocessed foods on health.

**Figure 1:**
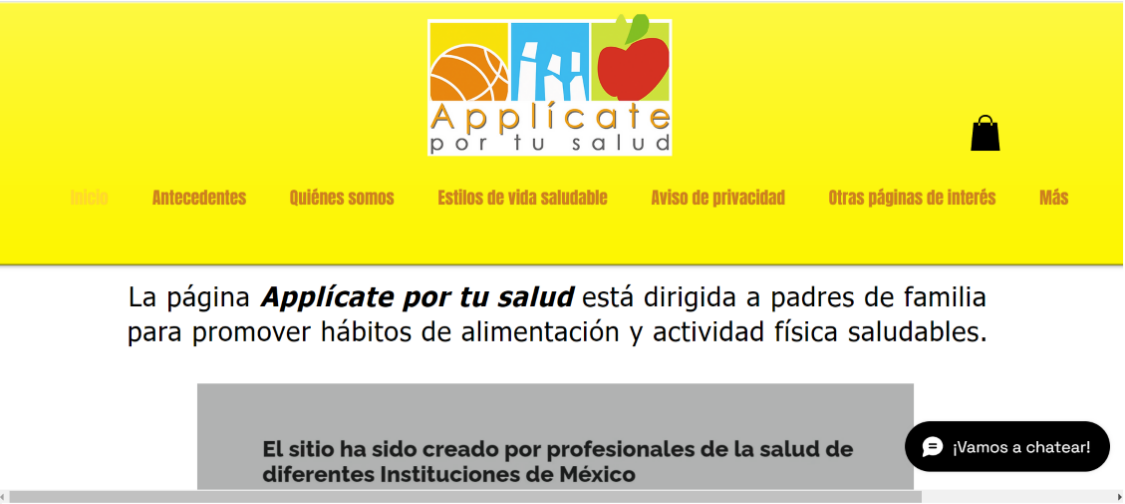
Website “Applicate por tu salud” (www.viviendo-saludable.org).

The intervention will be carried out via a website [23] (Figure 1) with audiovisual material, and all participants will also be asked to complete a multiple-choice evaluation (5 questions) to ensure a theoretical understanding of the topics. Other measures will include a lunch register, 24-hour dietary recall, a survey of food habits and consumption, a validated food preference questionnaire, anthropometric measurements (eg, weight, height, and waist circumference), a socioeconomic survey, as well as participation in a simulated web-based food and beverage selection and shopping activity. These measures will help assess if the digital educational intervention on front-of-package warning labeling for children and caregivers improves the selection and purchase of foods.

### Inclusion Criteria

Inclusion criteria for children are as follows: students in third, fourth, and fifth grades (aged 8 to 12 years), of both sexes, enrolled in the selected primary schools, with normal weight, overweight, and obesity, who sign the written informed consent.

Inclusion criteria for caregivers are as follows: primary caregivers of any sex of children in the third, fourth, and fifth grades with normal weight, overweight and obesity, who sign the written informed consent.

### Exclusion Criteria

Caregivers and children without internet access, computers, or mobile devices, as well as those participating in a weight reduction program with pharmacological treatment, will be excluded.

### Nutritional Education Interventions

The control group and the intervention group in dyads (caregiver-schoolchildren) will receive general nutritional education, and only the intervention group will receive guidance on reading warning labels, which raises awareness about the impact of consuming processed and ultraprocessed foods on health.

On the website, there will be 4 sections. Participants will receive an account and password to access the educational content corresponding to the group they belong, intervention or control group.

The intervention and control groups will have access to the first section, which contains general nutritional education topics. The second section will contain nutritional education topics related to warning labeling, and only the intervention group will have access to it.

Each topic will be presented through videos, released every 15 days, as well as weekly infographics and tips to the caregivers and children via the website. This material will also be sent to the participants’ mobile devices.

**Figure 2:**
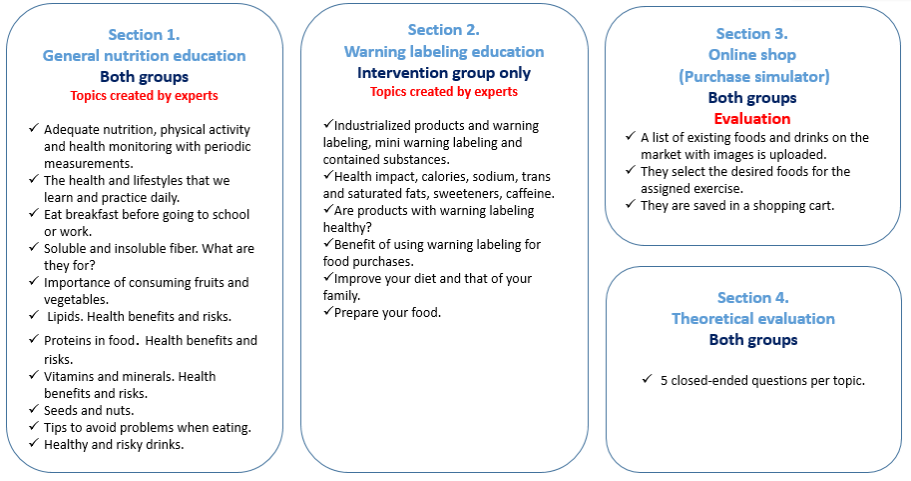
Nutritional intervention topics by groups.

The third section will have the “Online shop,” which will be used as part of the food selection evaluation. The fourth section will evaluate theoretical understanding with 5 multiple-choice questions per topic (Figure 2).

### Primary Outcomes

#### Time Frame

The measurements will be carried out in 2 scenarios (in schools and through the website) at baseline (1 week before starting the educational interventions), at 3 months, and at 6 months after the intervention ends.

A socioeconomic survey will be used in the baseline measurement.

The following assessments will be conducted at 3 frame times (baseline, 3 months, and 6 months): a food preference questionnaire, a 24-hour dietary recall, lunch register, and a survey of food habits and consumption. Anthropometric measurements (eg, weight, height, and waist circumference) will only be performed at baseline and the end of the 6-month intervention.

Through the website, dyads will engage in food and beverage purchasing exercises in the online shop simulator at the 3 measurement points (baseline, 3 months, and 6 months), along with multiple-choice evaluation (5 questions).

#### Food and Drink Selection

The percentage of products purchased by each participant in the online store, whose content indicates “high in calories, sugar, fat, and sodium” and the average content of these nutrients in 100 g of each product will be measured.

#### Energy and Macronutrient Intake

The amount of kilocalories consumed as well as saturated fat, trans fat, sodium, and added sugar will be measured.

### Statistical Analysis

Descriptive statistics will be used to characterize variables, and tables will be made with summary measures. Independent *t* tests and chi-square tests for categorical data will be used to compare baseline characteristics between the intervention and control groups. The Mann-Whitney *U* test will be used to evaluate differences between groups in case of nonnormal distribution.

Repeated measures ANOVA will be used to evaluate the change in the number of warning labels in nutrient consumption between groups and throughout the intervention. We will use a mixed-effects model to evaluate the effect of the intervention on changes in food and beverage selection, adjusting for confounding variables. Intention-to-treat analysis will be performed.

### Ethical Considerations

The protocol has obtained ethics approval by the bioethics committees of the Hospital Infantil de México Federico Gómez (HIM/2022/054). This protocol has also been registered with ClinicalTrials.gov (NCT06102473).

The objectives and activities of the study will be explained to the caregivers and children. To participate, they must sign the informed consent and informed assent forms to give their authorization to participate, with confidentiality guaranteed.

The website will contain health education material and an online shop to simulate the selection and shopping of food and beverages. No personal or medical information will be uploaded to the website. The results will only be used for the study and will be stored by the research team on password-protected computers. In case of any changes to the protocol, the ethics committee will be informed.

## Results

The protocol was registered in 2022 and was approved by the committees of the Federico Gómez Children's Hospital of Mexico, and they also obtained federal resources for the project. We expect to begin the study in schools in September 2024. We are not recruiting yet. The results will be analyzed and published in April and May 2025.

## Discussion

### Expected Outcomes

This protocol aims to study a public health policy in Mexico, which seeks to contribute to improving the health of the Mexican population by facilitating the understanding of nutritional information about ultraprocessed foods and beverages using front-of-package warning labeling. Studying the effects of this intervention on the population is essential for continuing with the initiative, improving it, or considering other measures to achieve the expected health outcomes.

Some studies have been conducted around the world on different types of front labeling, exploring their effectiveness in selecting and purchasing healthier foods.

A study on adults [24] explored the effectiveness of different types of frontal labeling. Participants were given a brief educational session through a smartphone app before being exposed to 2 experimental shopping tasks. The study found that all types of front labeling, after the educational intervention, resulted in better food selection. In Mexico, a study [25] provided guidance on front labeling via video before participants engaged in online food selection, showing positive effects on food selection.

Another study [26] demonstrated that nutritional warning labels with stamps significantly reduced the percentage of participants who selected products with excessive content of at least one nutrient, compared to the control group (62% vs 85%), and a statistically significant difference was observed for participants who selected products with an excess of certain nutrients, such as sugar (*P*<.001), saturated fats (*P*<.001), and sodium (*P*=.01).

A study in the United States [27] targeting parents concluded that warning labels on sugary beverages reduced the purchase of these products for their children. Although the evidence indicates that front-of-package warning labels can improve the selection of foods and beverages, the effects of these labels on the pediatric population and how children influence the selection and purchase of foods in their homes are still unknown.

On the other hand, the guidance received by participants in the aforementioned studies is limited to ensuring understanding of reading the label.

One limitation of the study is that the exercise of food and beverage selection will be made in a simulated shopping scenario, which may differ from real-life situations of food and beverage purchasing and consumption; however, this study is important to determine whether a structured educational intervention on key nutrients and raising awareness about their implication in health enhances the effects of front-of-package warning labeling to reduce overweight and obesity as well as other chronic diseases, such as diabetes and hypertension.

### Conclusions

It is expected that the study will contribute to evaluating whether reinforcing front-of-package warning labeling with education enhances its effects and makes them more sustainable. Carrying out this study will allow us to propose whether or not it is necessary to develop new intervention strategies related to front-of-package labeling for a better understanding of the population, improved food choices, and better health outcomes.

## Data Availability

The data sets generated and analyzed during this study are not publicly available to comply with the ethical requirements and participants’ confidentiality protection but are available from the corresponding author upon reasonable request.
